# Cellulosic ethanol production using a yeast consortium displaying a minicellulosome and β-glucosidase

**DOI:** 10.1186/1475-2859-12-14

**Published:** 2013-02-05

**Authors:** Sujin Kim, Seung-Ho Baek, Kyusung Lee, Ji-Sook Hahn

**Affiliations:** 1 School of Chemical and Biological Engineering, Seoul National University, 1 Gwanak-ro, Gwanak-gu, Seoul 151-744, Republic of Korea

**Keywords:** Cellulosic bioethanol, Cellulosome, Consolidated bioprocessing, Yeast surface display

## Abstract

**Background:**

Cellulosic biomass is considered as a promising alternative to fossil fuels, but its recalcitrant nature and high cost of cellulase are the major obstacles to utilize this material. Consolidated bioprocessing (CBP), combining cellulase production, saccharification, and fermentation into one step, has been proposed as the most efficient way to reduce the production cost of cellulosic bioethanol. In this study, we developed a cellulolytic yeast consortium for CBP, based on the surface display of cellulosome structure, mimicking the cellulolytic bacterium, *Clostridium thermocellum*.

**Results:**

We designed a cellulolytic yeast consortium composed of four different yeast strains capable of either displaying a scaffoldin (mini CipA) containing three cohesin domains derived from C. *thermocellum*, or secreting one of the three types of cellulases, *C. thermocellum* CelA (endoglucanase) containing its own dockerin, *Trichoderma reesei* CBHII (exoglucanase) fused with an exogenous dockerin from *C. thermocellum*, or *Aspergillus aculeatus* BGLI (β-glucosidase). The secreted dockerin-containing enzymes, CelA and CBHI, were randomly assembled to the surface-displayed mini CipA via cohesin-dockerin interactions. On the other hand, BGLI was independently assembled to the cell surface since we newly found that it already has a cell adhesion characteristic. We optimized the cellulosome activity and ethanol production by controlling the combination ratio among the four yeast strains. A mixture of cells with the optimized mini CipA:CelA:CBHII:BGLI ratio of 2:3:3:0.53 produced 1.80 g/l ethanol after 94 h, indicating about 20% increase compared with a consortium composed of an equal amount of each cell type (1.48 g/l).

**Conclusions:**

We produced cellulosic ethanol using a cellulolytic yeast consortium, which is composed of cells displaying mini cellulosomes generated via random assembly of CelA and CBHII to a mini CipA, and cells displaying BGLI independently. One of the advantages of this system is that ethanol production can be easily optimized by simply changing the combination ratio of the different populations. In addition, there is no limitation on the number of enzymes to be incorporated into this cellulosome structure. Not only cellulases used in this study, but also any other enzymes, including cellulases and hemicellulases, could be applied just by fusing dockerin domains to the enzymes.

## Background

Recently, there has been a growing interest in production of cellulosic bioethanol as a promising alternative to fossil fuels [1-3]. Cellulosic biomass is the most abundant materials in nature and relatively apart from the ethical concerns raised against the use of food-based materials such as corn and sugarcane [4,5]. Despite these advantages, however, the recalcitrant nature of cellulosic biomass and the high cost of cellulase are the major obstacles to utilize cellulosic biomass [6,7].

To completely degrade cellulose to fermentable glucose, the cooperative actions of at least three types of cellulases, endoglucanase, exoglucanase, and β-glucosidase, are required. Endoglucanase randomly breaks the β-1,4-glycosidic bonds within amorphous regions in crystalline cellulose. Exoglucanase further hydrolyzes the cellulose chains from their reducing or non-reducing ends, releasing cellodextrins, mainly cellobiose, which are finally converted to glucose by β-glucosidase [8,9].

Several anaerobic cellulolytic bacteria such as *Clostridium* and *Ruminococcus* species produce intricate multi-enzyme machines, termed cellulosome, that allow highly efficient cellulose degradation [10,11]. The assembly of cellulosome is mediated through structural scaffoldin that contains cohesin domains which interact with dockerin domains in enzymes. It has been generally considered that cellulosome complex leads to synergistic degradation of cellulosic substrate afforded by spatial proximity of the tethered cellulases. In addition, cellulose-binding module (CBM) in scaffoldin might also contribute to the cellulolytic efficiency via efficient substrate targeting [12].

Consolidated bioprocessing (CBP), combining cellulase production, saccharification, and fermentation into one step, has been proposed as the most efficient way to reduce the production cost of ethanol from cellulosic biomass [8,13]. In recent years, some efforts have been made to develop cellulolytic yeast strains for CBP by imitating the cellulosome structure [14-17]. Zhao group reported the first successful assembly of tri-functional minicellulosomes on the surface of *Saccharomyces cerevisiae*[14]. In their concept, three cellulases and a scaffoldin, containing a single type of cohesin and dockerin pair from *Clostridium thermocellum*, were co-expressed in a single strain, allowing a random assembly of the enzymes to the scaffoldin. However, expression of multiple genes in a single cell could cause metabolic burden and saturation of the cellular secretion system, which might restrict the efficiency of cellulosome assembly on the yeast surface.

On the other hand, Chen group constructed site-specific minicellulosomes using a scaffoldin carrying three divergent cohesin domains originated from three different strains, which allows site-specific binding of three enzymes, each tagged with the matching dockerin domain [16]. In addition, they introduced a consortium concept by expressing each cellulosome component, three cellulases and one scaffoldin, separately, and optimized the cellulolytic and ethanol production performance by adjusting the ratio of different populations in the consortium. Although site-specific interaction between cohesin and dockerin is capable of providing highly controllable ordering of each enzyme, there is a limitation as the ratio among the enzymes is fixed depending ultimately on the ratio of the corresponding cohesin. Moreover, such a site-specific assembly system would be disadvantageous for expanding the list of enzymes to be incorporated into the cellulosome complex.

Therefore, in this study, we combined the advantages of the consortium concept and the random assembly of cellulosome components. We designed a scaffoldin (mini CipA) composed of the same type of cohesins, which enables assembly of the dockerin-containing enzymes in a random manner. Since we newly found that β-glucosidase can bind to the yeast surface without any modification, endoglucanase and exoglucanase, but not β-glucosidase, were incorporated as cellulosome components. We optimized the cellulosome activity for ethanol production by controlling the combination ratio among the four yeast strains, capable of either displaying the mini CipA or secreting one of the three enzymes.

## Results and discussion

### Construction of a minicellulosome structure on the yeast surface

**Figure 1:**
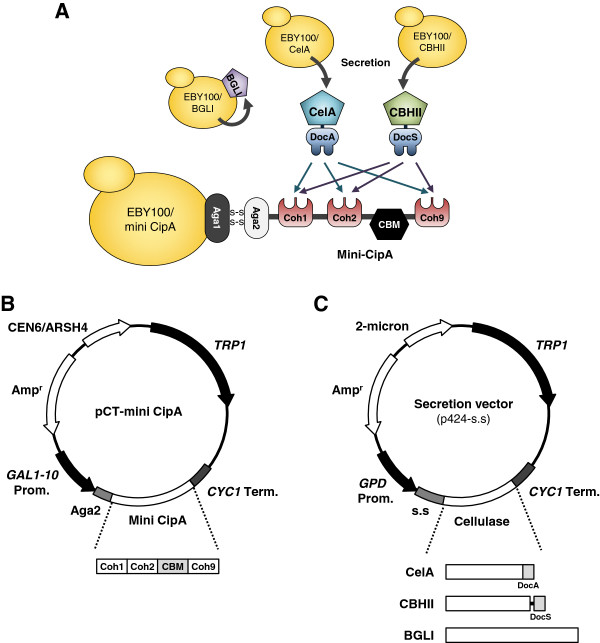
**Design of a minicellulosome structure on the yeast surface. (A) **Schematic diagram of the overall concept of this research. The basic design of this research is composed of four different yeast strains, one for displaying the mini CipA, and the others for secreting three types of cellulases. Mini CipA, a modified scaffoldin including a CBM and three cohesin domains, was expressed as an Aga2-fusion protein. The dockerin fused-enzymes, *C. thermocellum *CelA and *T. reesei *CBHII, were randomly assembled to mini CipA via cohesin-dockerin interactions, whereas the secreted *A. aculeatus *BGLI was independently bound to the cell surface through its own cell surface adhesion characteristic. Coh, cohesin; CBM, carbohydrate-binding module; DocA and DocS, native dockerin in CelA and exogenous dockerin from *C. thermocellum* CelS, respectively. **(B) **Construction of a surface display vector, pCT-mini CipA. Mini CipA, a modified scaffoldin containing a CBM and three cohesin domains, was expressed under the control of *GAL1 *promoter as an Aga2-fusion protein for displaying on the yeast surface. **(C) **Construction of cellulase secretion vectors. α-factor prepro-peptide (s.s) was used as a signal sequence.

The basic design of this research is composed of four different yeast strains, one of which for displaying the mini CipA, and the others for secreting one of the three types of cellulases (Figure 1A). Mini CipA, a modified *C. thermocellum* scaffoldin containing a CBM and three cohesin domains, was expressed under the control of *GAL1* promoter as an Aga2-fusion protein on the surface of yeast EBY100 strain (Figure 1B). In order to hydrolyze cellulose to glucose, endoglucanase CelA from *C. thermocellum*, exoglucanase CBHII from *Trichoderma reesei*, and β-glucosidase BGLI from *Aspergillus aculeatus* were secreted using α-factor prepro-peptide (Figure 1C). CelA and CBHII were expressed with the native dockerin (DocA) and exogenous dockerin from *C. thermocellum* CelS (DocS), respectively.

**Figure 2:**
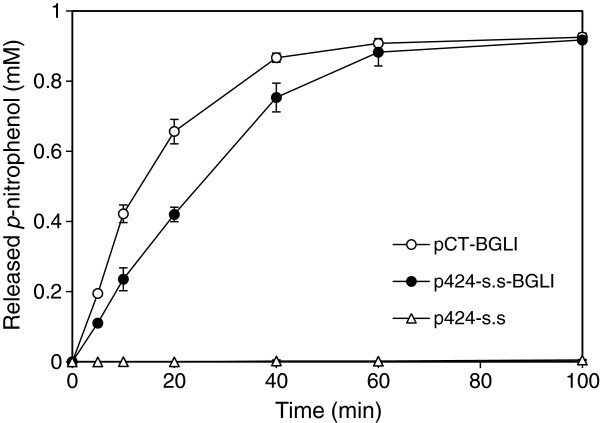
**Yeast surface adhesion characteristic of *A. aculeatus *BGLI. **The β-glucosidase activity was measured by pNPG assay using the harvested cells containing pCT-BGLI (BGL1 surface display vector, open circle), p424-s.s-BGLI (BGL1 secretion vector, closed circle), or p424-s.s (negative control, open triangle).

BGLI, on the other hand, was expressed without a dockerin domain since we found that it already has a cell adhesion characteristic without any additional anchor system. As shown in Figure 2, the harvested yeast cells harboring BGLI-secretion vector (p424-s.s-BGLI) showed β-glucosidase activity comparable to the cells containing pCT-BGLI, displaying BGLI on the surface as an Aga2-fusion protein. This observation might be in line with the cell wall-binding properties of the β-glucosidases reported in several fungi including *Aspergillus kawachii*, *Aspergillus oryzae*, *Neurospora crassa*, *Pichia etchellsii*, and *T. reesei*[18-22]. Therefore, in our study, dockerin fused-enzymes, CelA and CBHII, were randomly assembled to the scaffoldin structure via cohesin-dockerin interactions, whereas BGLI was assembled to the cell surface independently (Figure 1A).

### Cellulosic ethanol production using the optimized cellulosome

For complete degradation of pure cellulose to glucose, at least three different enzyme activities, including endoglucanase, exoglucanase, and β-glucosidase, are required [8]. However, each enzyme activity required for effective hydrolysis is depending on the types of enzymes and substrates, as well as the experimental conditions [23-26]. Therefore, we attempted to optimize the cellulosome activity by controlling the combination ratio among the yeast strains capable of either displaying the mini CipA or secreting one of the three enzymes.

**Figure 3:**
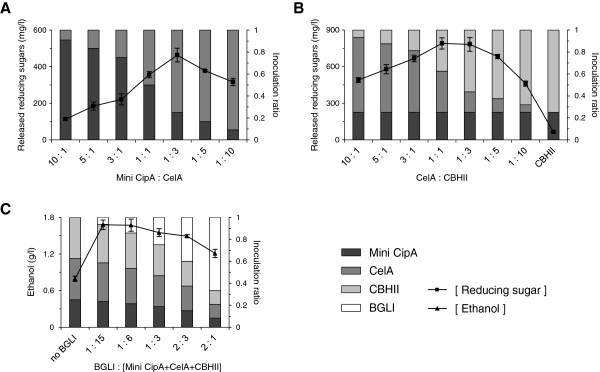
**Optimization of the cellulosome and ethanol production activity by controlling the combination ratio of different cell populations. (A) **The optimal ratio of mini CipA (cohesin) to CelA (dockerin). CelA was regarded as a representative of total cells harboring dockerin-fused enzymes. EBY100 cells displaying mini CipA and secreting CelA were combined at the indicated ratios, and the released reducing sugars from PASC were detected using the harvested cells. **(B) **The optimal ratio of CelA to CBHII. The optimal CelA:CBHII ratio for PASC hydrolysis was determined while fixing the amount of cells displaying mini CipA as 1/4 of total cells. **(C) **The optimal ratio of BGLI to cellulosome for ethanol production. The optimal amount of BGL1 for ethanol production was determined while fixing the mini CipA:CelA:CBHII ratio of 2:3:3. The bar graph represents inoculation portion of cells containing each plasmid. The square line graph represents released reducing sugars from PASC in each ratio. The triangle line graph represents ethanol concentration after three-day fermentation in each ratio.

First, in order to simplify possible combinations, we investigated the mini CipA portion of total population that makes the amount of displayed enzymes on the cell-surface maximum. EBY100 containing p424-s.s-CelA (EBY100/CelA) was regarded as a representative of cells secreting dockerin-fused enzymes. EBY100/CelA and EBY100 containing pCT-mini CipA (EBY100/mini CipA) were co-cultured for 24 h in synthetic galactose casamino acids (SG-CAA) medium in various ratios. The harvested cells were incubated with phosphoric acid swollen cellulose (PASC), and the released reducing sugars were measured by dinitrosalicylic acid (DNS) method. Detection of the CelA endoglucanase activity in the harvested cells indicates successful binding of the secreted CelA to the mini CipA displayed on the surface (Figure 3A). We also confirmed mini CipA-dependent display of CelA on the yeast surface by indirect immunofluorescence (data not shown). The amount of released reducing sugars reached a peak at mini CipA:CelA ratio of 1:3.

Thereafter, the ratio between EBY100/CelA and EBY100/CBHII was investigated while fixing the amount of EBY100/mini CipA as 1/4 of total cells. EBY100/CelA and EBY100/CBHII secreted similar amounts of proteins in the medium (data not shown). As shown in Figure 3B, the optimal ratio of EBY100/CelA:EBY100/CBHII, which produced the highest amounts of reducing sugars, was 1:1. Although CBHII alone did not show any significant PASC hydrolysis activity, the concentration of the released reducing sugars were higher in the presence of both CelA and CBHII (maximum 790 mg/l) than in the presence of CelA alone (maximum 460 mg/l), confirming the cooperative actions between the endoglucanase and exoglucanase.

Finally, we included EBY100/BGLI strain to the optimized minicellulosome system and measured ethanol production after three-day fermentation in 5 mL yeast extract-peptone-PASC (YP-PASC). Fixing the mini CipA:CelA:CBHII ratio of 2:3:3, we added EBY100/BGLI strain with various ratios. As shown in Figure 3C, BGLI:the other cells (mini CipA, CelA, and CBHII) in a ratio of 1:15 produced the highest concentration of ethanol. This result indicates that very small amount of BGLI, compared with other enzymes, is sufficient to completely hydrolyze the cellodextrins generated by CelA and CBHII. Taken together, the cellulosome activity showed the maximum performance at mini CipA:CelA:CBHII:BGLI in a ratio of 2:3:3:0.53.

**Figure 4:**
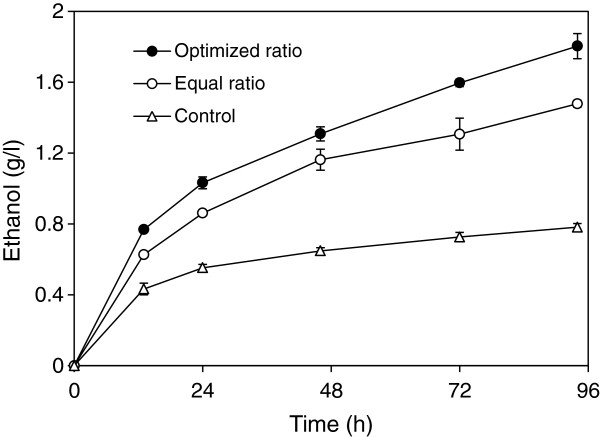
**Direct fermentation of amorphous cellulose to ethanol. **Time course of ethanol production from a yeast consortium composed of the mini CipA:CelA:CBHII:BGLI ratio of 2:3:3:0.53 (optimized ratio, closed circle) or 1:1:1:1 (equal ratio, open circle), or EBY100 cells containing pCT-mini CipA (control, open triangle).

The ability of direct ethanol fermentation from PASC was examined using a mixture of cells composed of the optimized ratio and an equal amount of each cell type. The maximum ethanol production was 1.80 g/l after 94 h in a consortium of the optimized ratio, which indicated about 20% increase compared with a consortium composed of an equal ratio (1.48 g/l) (Figure 4).

## Conclusions

In this study, we developed a novel cellulolytic yeast consortium for cellulosic ethanol production, which is composed of cells displaying mini cellulosomes generated via random assembly of CelA and CBHII to a mini CipA, and cells displaying BGLI independently. It is highly likely that the amount of each cellulase required for the maximum cellulolytic activity changes depending on the characteristics of target substrate and each cellulase. Therefore, one of the advantages of this system is the convenient optimization of ethanol production by simply changing the combination ratio of the different populations. In addition, not only cellulases used in this study, but also any other enzymes, including cellulases and hemicellulases, could be applied just by fusing dockerins to the enzymes. It means that there is no limitation on the number of cellulases to be incorporated to the cellulosome structure. We also found that *A. aculeatus* BGLI can bind to yeast cell surface without any modification, which should be considered in interpreting or designing cellulolytic yeast system involving secreted BGLs originated from fungi.

## Methods

### Strains and media

*Escherichia coli* strain DH5α [F^-^ Φ*80lacZ*Δ*M15* Δ(*lacZYA-argF*) *U169 recA1 endA1 hsdR17 (r*_*K*_^*–*^*, m*_*K*_^*+*^*) phoA supE44* λ^*–*^*thi-1 gyrA96 relA*] was used for genetic manipulations. *S. cerevisiae* strain EBY100 (*MAT*a *GAL1-AGA1::URA3 ura3-52 trp1 leu2*Δ*1 his3*Δ*200 pep4::HIS2 prb1*Δ*1.6R can1 GAL*) was used for surface display of scaffoldin or cellulase secretion. *E. coli* was cultured in Luria-Bertani (LB) medium (10 g/l tryptone, 5 g/l yeast extract, and 10 g/l NaCl) supplemented with 50 μg/ml ampicillin. *S. cerevisiae* EBY100 transformants containing plasmids were grown in synthetic dextrose casamino acids (SD-CAA) medium (20 g/l dextrose, 6.7 g/l yeast nitrogen base, 5 g/l casamino acids, 5.4 g/l Na_2_HPO_4_, and 8.6 g/l NaH_2_PO_4_·H_2_O). For the induction of scaffoldin cloned in pCTCON vector, cells grown in SD-CAA medium were harvested and resuspended in SG-CAA medium containing galactose instead of glucose, and further incubated for 24 h at 30°C.

### Plasmid construction

Plasmid pCT-mini CipA for surface display of scaffoldin was constructed as follows. The DNA fragments encoding amino acids 28–559 (1.6 kb, two cohesin domains and CBM) and 1533–1698 (0.5 kb, a cohesin domain) of the *C. thermocellum* CipA were prepared by PCR amplification, using genomic DNA (ATCC 27405) as template. These PCR products were sequentially cloned into the restriction sites SacI/SpeI and SpeI/XhoI of pRS426GPD vector [27], and then amplified by using PCR primers, each containing 35 nt or 37 nt region homologous to the pCTCON vector. PCR product was transformed into *S. cerevisiae* EBY100 together with the NheI and BamHI digested pCTCON vector, leading to an insertion of the PCR product into a pCTCON vector by homologous recombination. The resulting plasmid, pCT-mini CipA was isolated from the transformant and confirmed by DNA sequencing. Plasmid p424-s.s for the secretion of cellulase was constructed as follows. The DNA fragment encoding secretion signal sequence from the *S. cerevisiae* α-factor prepro-peptide was prepared by PCR amplification, using pPICZαA as template and primers containing additional restriction enzyme recognition sites (ApaI, BamHI, BglII and NheI). The 330 bp PCR product was cloned into the SpeI and SalI sites of pRS424GPD vector resulting in p424-s.s vector. *C. thermocellum* CelA gene lacking signal sequence was amplified from genomic DNA (ATCC 27405) and cloned into the ApaI/NheI sites of p424-s.s vector. The dockerin domain of *C. thermocellum* CelS (DocS) was amplified and cloned into the NheI and SalI sites of p424-s.s vector. *T. reesei* CBHII and *A. aculeatus* BGLI genes lacking signal sequence were amplified from cDNA containing vectors [28] and cloned into the ApaI/NheI sites of p424-s.s-DocS and BamHI/NheI sites of p424-s.s vector, respectively*.*

### β-glucosidase activity assay

The activity of β-glucosidase BGL1 was determined by measuring the amount of *p*-nitrophenol released from *p*-nitrophenyl-β-D-glucopyranoside (*p*NPG) used as substrate. A_600_ of 10 cells were washed twice with distilled water and twice with reaction buffer (50 mM sodium acetate, pH 5.2). Cells were resuspended in a reaction buffer containing 1 mM *p*NPG and incubated at 30°C. The amount of hydrolyzed *p*-nitrophenol was quantified by measuring the optical density at 400 nm using 96-well plate spectrophotometer (Multiskan GO, Thermo Scientific, USA).

### Endo/exoglucanase activity assays

Yeast cells, each harboring the surface-display plasmid for mini CipA or secretion plasmid for CelA or CBHII were grown in SD-CAA medium and then mixed in various combinations in SG-CAA medium for 24 h. A_600_ of 10 of cells were harvested and washed with reaction buffer (50 mM sodium acetate, pH 5.2). Cells were resuspended in a reaction buffer containing 1% PASC and then incubated for 4 h at 30°C. The amount of reducing sugars in the reaction supernatant was determined by DNS method as described elsewhere [29].

### Fermentation

A_600_ of 50 of cells were harvested and resuspended in YP-PASC medium (10 g/l yeast extract, 20 g/l peptone, and 1% PASC as the sole carbon source). Ethanol fermentation was carried out as previously described with minor modification [28]. Cell mixture was anaerobically incubated in a 30 ml-serum bottle with 10 ml reaction volume. High performance liquid chromatography (HPLC) (Finnigan Surveyor Plus, Thermo Scientific, USA) was used to quantify ethanol concentration. HPLC was operated using a BioRad Aminex HPX-87H column (300 mm × 7.8 mm, 5 μm) at 60°C with 5 mM H_2_SO_4_ as eluent at a flow rate of 0.8 ml/min. Refractive index (RI) detector (Finnigan Surveyor RI Plus detector, Thermo Scientific, USA) was used to determine the ethanol quantification.

## Abbreviations

CBM: Carbohydrate-binding module; CBP: Consolidated bioprocessing; SG-CAA: Synthetic galactose casamino acids; DNS: Dinitrosalicylic acid; PASC: Phosphoric acid swollen cellulose; YP: Yeast extract-peptone; LB: Luria-Bertani; SD-CAA: Synthetic dextrose casamino acids; *p*NPG: *p*-nitrophenyl-β-D-glucopyranoside; HPLC: High performance liquid chromatography; RI: Refractive index.

## Competing interests

The authors declare that they have no competing interests.

## Authors’ contributions

SK and JH designed the experiments and wrote the manuscript. SK and SB performed the experiments. SK and KL analyzed the data. All authors read and approved the final manuscript.
